# High‐Efficiency and Stable Long‐Persistent Luminescence from Undoped Cesium Cadmium Chlorine Crystals Induced by Intrinsic Point Defects

**DOI:** 10.1002/advs.202207331

**Published:** 2023-02-24

**Authors:** Ruoting Yang, Dongwen Yang, Meng Wang, Fei Zhang, Xinzhen Ji, Mengyao Zhang, Mochen Jia, Xu Chen, Di Wu, Xin Jian Li, Yu Zhang, Zhifeng Shi, Chongxin Shan

**Affiliations:** ^1^ Key Laboratory of Materials Physics of Ministry of Education School of Physics and Microelectronics Zhengzhou University Daxue Road 75 Zhengzhou 450052 P. R. China; ^2^ State Key Laboratory on Integrated Optoelectronics College of Electronic Science and Engineering Jilin University Qianjin Street 2699 Changchun 130012 P. R. China

**Keywords:** cesium cadmium chloride, de‐trapping, information storage, long‐persistent luminescence, stability

## Abstract

Application of long‐persistent luminescence (LPL) materials in many technological fields is in the spotlight. However, the exploration of undoped persistent luminescent materials with high emission efficiency, robust stability, and long persistent duration remains challenging. Here, inorganic cesium cadmium chlorine (CsCdCl_3_) is developed, featuring remarkable LPL characteristics at room temperature, which is synthesized by a facile hydrothermal method. Excited by ultraviolet light, the CsCdCl_3_ crystals exhibit an intense yellow emission with a large photoluminescence quantum yield of ≈90%. Different from the reported systems with lanthanides or transition metals doping, the CsCdCl_3_ crystals without dopants perform yellow LPL with a long duration of 6000 s. Joint experiment‐theory characterizations reveal the intrinsic point defects of CsCdCl_3_ act as the trap centers of excited electrons and the carrier de‐trapping process from such trap sites to localized emission centers contributes to the LPL. Encouraged by the attractive fluorescence and persistent luminescence as well as good stability of CsCdCl_3_ against environment oxygen/moisture (75%), heat (100 °C for 10 h), and ultraviolet light irradiation, an effective dual‐mode information storage‐reading application is demonstrated. The results open up a new frontier for exploring LPL materials without dopants and provide an opportunity for advanced information storage compatible for practical applications.

## Graphical abstract

A long‐persistent luminescence material is developed based on undoped metal halide CsCdCl_3_ synthesized by hydrothermal approach. The persistent luminescence is observed at room temperature with a long duration of 6000 s. Moreover, the CsCdCl_3_ crystals exhibit excellent stability against environment oxygen/moisture, heat and ultraviolet light. Finally, successful applications of such long‐persistent luminescence material in dual‐mode information storage‐reading are demonstrated.

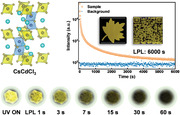

## 1 Introduction

Long‐persistent luminescence (LPL) materials that still emit light after ceasing the excitation source have acquired enduring research interest owing to their fundamental scientific importance and versatile applications.^[^ ^1^, ^2^, ^3^, ^4^ ^]^ Generally, traps and emitters as active centers are related to the occurrence of LPL. The former determines the afterglow performances including the persistent emission intensity and decay time, while the latter determines the emission wavelength. In host matrices, the defects‐related traps store excitation energy and then release it to the emitters under external stimulation. Since its first discovery in the early 20th century, a large number of persistent luminescence materials have been explored by changing host matrices with different crystal structures and dopants with different combinations of transition metals or lanthanides.^[^ ^5^, ^6^ ^]^ Among them, materials with activators doped in different oxide hosts (aluminates, silicates, stannate, etc.)^[^ ^7^, ^8^, ^9^ ^]^ exhibit excellent LPL performance, and the corresponding spectral range covers ultraviolet (UV), visible, and even near‐infrared light.^[^ ^10^, ^11^, ^12^ ^]^ But the high lattice‐formation energy (≈3000 kJ mol^−1^) of such materials results in the requirement of high synthesis temperature for phase generation,^[^ ^13^ ^]^ leading to an increase in manufacturing costs and safety risks.^[^ ^14^, ^15^, ^16^ ^]^ Moreover, a higher synthesis temperature would induce agglomerated products with irregular morphology with strong scattering effect, limiting the signal collection.^[^ ^17^, ^18^, ^19^ ^]^ Besides, organic LPL materials are also constantly being explored,^[^ ^20^ ^]^ which can be easily synthesized without the need for high temperatures.^[^ ^21^ ^]^ While traditional organic LPL materials always have shorter lifetimes at room temperature (RT) compared with the inorganic counterparts.^[^ ^22^ ^]^ To prolong the charge transfer transition of organic LPL materials, many effective strategies have been developed, such as engineering crystallization,^[^ ^23^, ^24^ ^]^ host‐guest interactions,^[^ ^25^, ^26^, ^27^ ^]^ heavy metal doping,^[^ ^28^ ^]^ and hydrogen bond interactions.^[^ ^24^, ^29^ ^]^ Although rapid progresses in this field have been witnessed in recent years, some intrinsic disadvantages of organic LPL materials cast a gloomy shadow for their further practical applications.^[^ ^30^, ^31^, ^32^, ^33^ ^]^ For instance, the organic molecules are very susceptible to air environment; oxygen and moisture would cause quenching of triplet excitons.

In the past several years, metal‐halide perovskites have gained increasing attention by virtue of their great potential in optoelectronic applications.^[^ ^34^, ^35^, ^36^, ^37^, ^38^, ^39^, ^40^, ^41^ ^]^ Such materials of both low lattice‐formation energy and versatile doping sites could enable a low‐temperature synthesis of LPL crystals.^[^ ^42^ ^]^ In the past two years, much interesting progress on LPL characteristics of perovskites has been reported successively. For instance, Xu et al. prepared the CsPbBr_3_:Ln^3+^ nanocrystals with high color‐purity LPL via a melt‐quenching process, which creates the carrier capture centers by replacing Pb^2+^ with Ln^3+^.^[^ ^43^ ^]^ Zhang et al. prepared the Cs_2_Na_x_Ag_1‐x_InCl_6_:Mn^2+^ and Cs_2_Na_0.2_Ag_0.8_InCl_6_:Yb^3+^ single crystals (SCs) via a simple hydrothermal process, exhibiting excellent orange and near‐infrared LPL, respectively.^[^ ^13^, ^42^ ^]^ Further, they employed a similar method to grow Tb^3+^‐doped Cs_2_NaScCl_6_ SCs at 180 °C, which exhibit a bright green LPL after ceasing the X‐ray excitation.^[^ ^44^ ^]^ The above results suggest the LPL performances achieved from metal‐halide perovskites are promising. However, it is an indisputable fact that the reported LPL from metal‐halide perovskites is all realized by creating carrier capture centers in matrix with a strategy of rare‐earth ions doping.^[^ ^45^ ^]^ From an application perspective, the realization of considerable LPL in pristine perovskite systems without dopants is valuable and irresistible, considering the following three aspects mainly. First, the distribution of traps in hosts is sensitive to the concentration of ion doping, and the controllability of trap engineering is controversial. Second, ion doping requires higher activation energy to be entered into the matrix lattice, increasing the requirement for material synthesis. Finally, as mature activators for persistent luminescence, rare‐earth ions are rare strategic resources, and their inefficient doping process is not conducive to the rational allocation of resources.

In this work, a high‐performance LPL material was developed based on undoped inorganic metal‐halide CsCdCl_3_, which was synthesized by a facile hydrothermal approach. Under UV light (254 nm) excitation, the synthesized CsCdCl_3_ crystals exhibit a broadband yellow emission related with the self‐trapped excitons formed in Cd—Cl octahedron, and the photoluminescence quantum yield (PLQY) is as high as ≈90%. Excitingly, a persistent luminescence was observed at RT with a long duration time of 6000 s. A combination of experimental characterizations together with theoretical calculations identifies the carrier de‐trapping mechanisms from shallow and deep trap sites induced by the intrinsic point defects of CsCdCl_3_. Importantly, CsCdCl_3_ SCs exhibit excellent environmental, thermal, and light stability. Enlightened by the present results, we further demonstrate the applications of such LPL material in dual‐mode information storage‐reading. This study offers an example of the development of LPL materials without dopants and opens up avenues for advanced applications in information storage.

## 2 Result and Discussion

**Figure 1:**
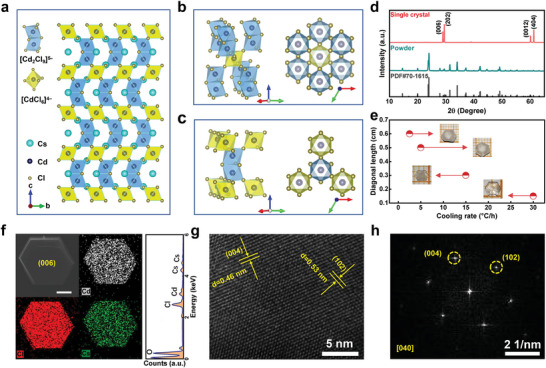
a) Crystal structure of CsCdCl_3_ in the [001] direction. The blue unit is [Cd_2_Cl_9_]^5−^ dimers and the yellow unit is [CdCl_6_]^4−^ octahedron. b) Bonding diagram of an octahedron to its surrounding units. c) Bonding diagram of a dimer to its surrounding units. d) XRD patterns of the CsCdCl_3_ SCs and ground powder. e) Relationship between the diagonal length of CsCdCl_3_ SCs and the cooling rate. f) SEM image of a CsCdCl_3_ crystal and the corresponding elemental mappings. The scale bar is 500 µm. g) High‐resolution TEM image of CsCdCl_3_ SC. h) The corresponding FFT image.

**Figure** **1**a displays the schematic crystal structure of CsCdCl_3_, showing unique octahedral bonding, in which [Cd_2_Cl_9_]^5−^ subunits formed with two face‐sharing octahedra share corners with independent [CdCl_6_]^4−^ octahedron, thus unfold in three dimensions.^[^ ^46^, ^47^ ^]^ Specifically, each [CdCl_6_]^4−^ octahedron shares corners with six [Cd_2_Cl_9_]^5−^ dimers, and each [Cd_2_Cl_9_]^5−^ dimer shares corners with six [CdCl_6_]^4−^ octahedra (Figure 1b,c). Figure 1d shows the typical X‐ray diffraction (XRD) patterns of CsCdCl_3_ samples (SCs and the corresponding ground powders). One can see that the detected diffraction peaks for CsCdCl_3_ SCs and ground powders are all consistent with the standard card of hexagonal CsCdCl_3_ (PDF# 01‐070‐1615), and no additional diffraction peaks emerge, which suggests that there are no impurities inside the prepared CsCdCl_3_ crystals. The detailed crystal structure data and atomic positions of hexagonal CsCdCl_3_ were summarized in Tables S1 and S2, Supporting Information, respectively. Figure S1, Supporting Information, displays the Raman spectra of CsCdCl_3_ SCs and ground powder. The measured spectra are characterized by similar features with four peaks. The mode of 69 cm^−1^ is related to the *E*_2g_, and the peak at 115, 129, and 248 cm^−1^ are attributed to *A*_1g_ modes of CsCdCl_3_.^[^ ^47^, ^48^ ^]^ This result further excludes the possibility that small domain of impurities is embedded in the crystal. X‐ray photoelectron spectroscopy (XPS) tests were conducted to examine the chemical valence of CsCdCl_3_ crystals. Figure S2, Supporting Information, presents a survey XPS spectrum, and the signals attributed to Cs, Cd, and Cl were obtained. Specifically, the Cd 3d spectrum has well‐separated spin‐orbit components, and the bands peaked at 410.9 and 404.2 eV are related to the Cd 3d_3/2_ and Cd 3d_5/2_, respectively. Two separate peaks at 736.5 and 722.5 eV are assigned to the Cs 3d_3/2_ and Cs 3d_5/2_, and two distinct peaks observed at 198.2 and 196.8 eV are associated with the 2p_1/2_ and 2p_3/2_ of Cl^−^, well consistent with the previous studies.^[^ ^49^, ^50^ ^]^ Figure 1e presents the dependence of diagonal length of CsCdCl_3_ SCs on the cooling rate, and a monotonically decreasing trend was revealed. Within a cooling rate window of 2.5–30 °C h^−1^, the diagonal length of CsCdCl_3_ SCs can be adjusted from 0.15 to 0.6 mm, while the colorless and transparent hexagonal shape was maintained well. Such size evolution can be ascribed to the reduced supersaturation at low cooling rate, which is conducive to promoting the nucleation and growth of the crystals. Figure 1f displays the typical morphology of the prepared CsCdCl_3_ SC, showing a smooth and flat surface with regular hexagonal shape. In combination with the XRD results of CsCdCl_3_ and the corresponding reciprocal lattice, we confirm that the hexagonal exposed facet in the scanning electron microscope (SEM) image is (006) crystal plane. From the corresponding energy dispersive spectroscopy (EDS) elemental mapping, one can observe that Cs, Cd, and Cl elements are homogeneously distributed throughout the crystal. Based on the results of EDS, the quantitative atomic ratio (%) of Cs:Cd:Cl is determined to be 0.86:0.77:2.68, which is consistent with the stoichiometry of CsCdCl_3_ (Table S3, Supporting Information). Clear lattice fringes can be observed in the high‐resolution transmission electron microscopy (TEM) image of CsCdCl_3_ SC in Figure 1g, corresponding to the (004) and (102) crystal planes of hexagonal CsCdCl_3_. Figure 1h displays the fast Fourier transformation (FFT) image, which obviously demonstrates the excellent crystallization of the CsCdCl_3_ crystals.

**Figure 2:**
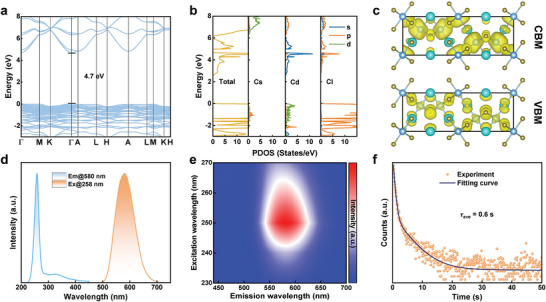
a) The calculated band structure and b) the corresponding PDOS of CsCdCl_3_. c) The isosurface plots of the wave function |Ψ|^2^ of CBM and VBM. d) PL and PLE spectra of CsCdCl_3_ SCs. e) Pseudocolor map of excitation‐dependent PL spectra of the CsCdCl_3_ SCs. f) Time‐resolved PL decay curves monitored under 254 nm excitation in the time window of 50 s.

Further, we investigated the electronic band structure and projected density of states (PDOS) of CsCdCl_3_ on the basis of the first‐principles calculations. **Figure** **2**a exhibits the calculated band structure, which has a direct bandgap of 4.7 eV. The PDOS suggests that the conduction band of CsCdCl_3_ is mainly composed of Cl 3p and Cd 5s orbitals, and the valence band mostly originates from the Cl 3p and Cd 5d states, and Cs cation displays negligible influence to the frontier orbitals (Figure 2b). Correspondingly, the charge density concentrates in Cd—Cl polyhedrons rather than Cs atoms analyzed by the wave functions |Ψ|^2^ of conduction band minimum (CBM) and valence band maximum (VBM), which are clearly depicted in Figure 2c. That is, the carriers prefer to be located in the Cd—Cl polyhedron in the lattices of CsCdCl_3_.

We further studied the optical properties of CsCdCl_3_ SCs experimentally. The UV–visible absorption spectrum of CsCdCl_3_ SCs exhibits an absorption peak located at 255 nm, and the corresponding Tauc plot exports a direct bandgap of 4.74 eV, close to the calculated data described above (Figure S3, Supporting Information). As shown in Figure 2d, under UV light excitation, the CsCdCl_3_ SCs display a broad yellow emission that peaked at 580 nm, and the full‐width at half‐maximum (FWHM) of the PL spectrum is ≈81 nm. The pale‐blue line shows a PL excitation (PLE) spectrum of CsCdCl_3_ SCs peaked at 258 nm. The broadband emission and the large Stokes shift (322 nm) suggest that the PL mechanisms are likely to result from the self‐trapped excitons which are always reported in low‐dimensional perovskites rather than the direct band‐edge transition.^[^ ^51^, ^52^, ^53^, ^54^ ^]^ Figure 2e presents the pseudocolor map of wavelength‐dependent PL and PLE spectra. The peak position and band shape of the PL spectra are almost independent of different excitation and emission wavelengths monitored, implying that the broadband yellow emission is derived from an excited state rather than the superposition of many defects‐related carrier recombination centers. Figure S4, Supporting Information, shows the excitation power‐dependent PL spectra of CsCdCl_3_, and the PL intensity is linearly dependent on the excitation power, excluding the emission that originates from the defect centers. Besides, the measured absolute PLQY of the CsCdCl_3_ crystals is ≈90% (Figure S5, Supporting Information), manifesting an outstanding emission property of products. The relationship of integrated PL intensity and reciprocal temperature is plotted to calculate the exciton binding energy of CsCdCl_3_ crystals, and the exciton binding energy of CsCdCl_3_ was calculated to ≈603.33 meV (Figure S6, Supporting Information). Such a large value is similar to that of the Cu‐based halide SCs with self‐trapped excitons nature,^[^ ^55^, ^56^ ^]^ which can guarantee the generation of excitons at RT and promote their radiative recombination.^[^ ^57^ ^]^ Figure 2f presents the PL decay curve monitored at 580 nm excited by 254 nm light, which is fitted with biexponential decay function (*R* ^2^ = 0.99), and the average PL lifetime is estimated as 0.6 s. Such a long PL lifetime is attributed to the LPL character of the CsCdCl_3_ sample, which will be explained in more detail later.^[^ ^13^ ^]^

**Figure 3:**
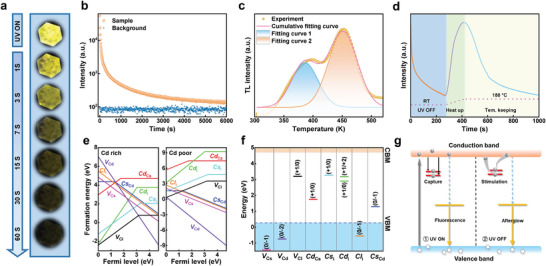
a) Fluorescence and LPL photographs of one CsCdCl_3_ SC. b) LPL decay curve monitored at 580 nm after stopping UV excitation (254 nm). c) Thermally stimulated luminescence spectrum of CsCdCl_3_ SC measured from 300 to 520 K. The heating rate is 1 K s^−1^. d) Luminescence profile of CsCdCl_3_ SC after cessation of excitation, followed by the thermal stimulation (the material was first charged using an UV lamp, while the emission signal was then monitored after turning off the lamp; after 270 s, the sample was heated to 180 °C). e) Function diagram of the formation energies of the point defects at different Fermi levels. f) Transition energy levels of intrinsic defects in CsCdCl_3_. g) Configuration coordinate diagram for the LPL mechanisms of CsCdCl_3_.

Interestingly, the CsCdCl_3_ SC exhibits a long‐term yellow LPL after ceasing the UV irradiation (**Figure** **3**a), which can be observed by naked eyes for one minute. Figure S7, Supporting Information, presents the time‐resolved LPL spectra recorded at different time periods after turning off the excitation light, showing an ever‐reducing peak intensity with a fixed peak position at 580 nm. The normalized LPL and PL spectra show that there is almost no difference in spectral shape or peak position (Figure S8, Supporting Information), implying the persistent luminescence originates from the same emission center to fluorescence in CsCdCl_3_. Further, the LPL decay curve of CsCdCl_3_ was recorded at RT to recognize the persistent luminescence character. As seen in Figure 3b, the LPL intensity drops abruptly in the first 150 s, followed by an ultra‐slow decay. The duration of LPL signal is up to 6000 s, remaining a signal level of two times larger than the noise level. It is worth mentioning that this duration time is longer than that of many non‐doping LPL systems, such as SrAl_2_O_4_ reported by Huang et al., Mg_2_SnO_4_ prepared by Hu et al., and Cs_3_In_2_Cl_9_ reported by Chen et al.^[^ ^45^, ^58^, ^59^ ^]^ Moreover, the duration of LPL in the present case is much higher than many doped metal halide systems,^[^ ^60^, ^61^ ^]^ demonstrating the application prospect of pristine CsCdCl_3_ as a promising LPL candidate.

Although the research on LPL has been going on for years, the mechanisms of the LPL phenomenon are still controversial. In general, the LPL phenomenon is closely related to the intrinsic or extrinsic defects that act as the trapping centers in the matrix.^[^ ^62^ ^]^ To examine the distribution of active afterglow traps, the thermoluminescence (TL) spectroscopy was therefore measured.^[^ ^63^ ^]^ As shown in Figure 3c, the measured TL curve is a broad spectrum with two TL peaks obtained by Gaussian fitting at 385 and 451 K, respectively, suggesting that there are two kinds of trap sites (shallow traps and deep traps) that continuously distributed in the CsCdCl_3_ SC.^[^ ^12^, ^64^ ^]^ In addition, the light intensity‐time curve (*I* ^−1^–*t*) converted from the LPL decay curve cannot be fitted to a straight line (Figure S9, Supporting Information), implying that the trapped carriers are returned to the conduction band by the thermal de‐trapping process.^[^ ^11^, ^65^ ^]^ Evidently, the shallow traps play a major role in LPL at RT, while the contribution of deep traps is negligible because a high activation energy is needed to overcome the energy barrier to enable the de‐trapping process of electrons. To prove this, a thermal stimulation treatment was therefore conducted to activate the deep trap sites, facilitating the de‐trapping process of electrons. As shown in Figure 3d, after the first LPL decay for 270 s (stage I), the sample is rapidly heated to 180 °C within 45 s (stage II), during which the LPL intensity increases abruptly, which could be assigned to the de‐trapping process of electrons in the deep traps that gain enough energy at high temperatures, implying the excellent energy storage capability of CsCdCl_3_ SC. During the subsequent temperature‐keeping stage (stage III) at 180 °C, the second LPL decay occurs. Obviously, two LPL decay behaviors at RT and 180 °C come from different electron de‐trapping processes, one from shallow trapping centers and another from deep trapping centers.^[^ ^66^ ^]^ To further demonstrate that thermal stimulation can activate deep trap sites, PL spectra at different temperatures were characterized (Figure S10, Supporting Information). At high temperatures, the electrons in the deep traps enable to escape and transfer to Cd—Cl octahedra to participate in emission, which overcomes the influence of thermal‐related non‐radiative recombination and results in a slight increase in PL intensity.

To further explore the formation of trapping centers in CsCdCl_3_ SC, the first‐principles calculations are conducted to study energetics and transition energy levels of intrinsic defects, in which three vacancy defects (*V*_Cs_, *V*_Cd_, and *V*_Cl_), three interstitial defects (*Cs*_i_, *Cd*_i_, and *Cl*_i_), and two cation substitution defects (*Cs*_Cd_ and *Cd*_Cs_) are considered. Two chemical potentials, Cd‐poor and Cd‐rich, are chosen to perform the calculations, due to the formation energy of a point defect is greatly affected by the chemical potential of component elements. As shown in Figure 3e, eight defects in CsCdCl_3_ are considered to calculate their formation energies at different Fermi level, in which the formation energies of *V*_Cl_ and *Cs*_i_ at Cd‐rich conditions are lower than other defects, implying that both types of defects are easily formed in hexagonal CsCdCl_3_. As the condition transits to Cd‐poor, the formation energy of *V*_Cd_ is the lowest. Further, we calculated the energy level positions of the intrinsic defects in CsCdCl_3_. As illustrated in Figure 3f, the *V*_Cl_ and *Cs*_i_ defects could create relatively shallow energy levels within the bandgap, so they could act as the charge trap states in CsCdCl_3_. While, the energy level of *V*_Cd_ defect does not locate within the bandgap of CsCdCl_3_, thus it could not be involved in the persistent emission even if its formation energy is rather low. Therefore, we consider that the intrinsic defects of *V*_Cl_ and *Cs*_i_ in CsCdCl_3_ SC form electron trapping centers near the conduction band, contributing to the LPL. Based on the above discussions, we illustrated the proposed LPL mechanisms of CsCdCl_3_ in Figure 3g. The entire LPL process is divided into two parts based on the UV lamp turning on or off. First, in the valence band, the electrons promoted by UV excitation are transformed from ground state to the excited state. After a period of relaxation, part of free electrons in the conduction band fall into the lower energy luminescent center and then recombine with holes in the valence band, emitting yellow fluorescence; another part of the electrons is captured and stored by the trapping centers close to the conduction band. Second, after the light excitation is stopped, the electrons stored in the traps are continuously released back to the conduction band under external stimuli (thermal de‐trapping), and subsequently captured by the luminescent center to emit yellow LPL.

**Figure 4:**
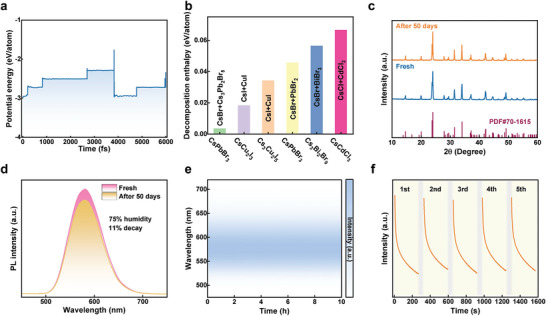
a) Potential energy fluctuations of CsCdCl_3_ as a function of the molecular dynamic simulation step at 300 K. b) The calculated decomposition enthalpies of CsCdCl_3_ and other inorganic halide perovskites. c) XRD patterns, and d) PL spectra of the CsCdCl_3_ powder before and after storage for 50 days in air ambient (75% humidity). e) The evolutional PL spectra of a CsCdCl_3_ SC under continuous heating at 100 °C for 10 h. f) Successive five‐cycled LPL decay curves of CsCdCl_3_ SC. The gray background corresponds to the time of heating to release the stored energy.

The stability of LPL materials is a crucial parameter to evaluate the reliability of their practical applications.^[^ ^1^ ^]^ Herein, combining the theoretical calculations and experimental characterizations, we evaluate the stability of the as‐prepared CsCdCl_3_ SCs. Figure S11, Supporting Information, shows the calculated phonon band structures of CsCdCl_3_, and no virtual frequencies were observed, verifying the dynamic stability of CsCdCl_3_. **Figure** **4**a presents the variation diagram of the thermodynamic stability of CsCdCl_3_ with time at 300 K, and a small potential energy fluctuation manifests it have a fine thermodynamic stability. Furthermore, the calculations are extended to the decomposition enthalpy. As summarized in Figure 4b, the calculated decomposition enthalpy of CsCdCl_3_ is much larger than other all‐inorganic metal halides counterparts (CsPbBr_3_, CsCu_2_I_3_, Cs_3_Cu_2_I_5_, and Cs_3_Bi_2_Br_9_), indicating the better stability of CsCdCl_3_ and opening up huge opportunities for future practical applications. The high structural stability of CsCdCl_3_ may be due to the face‐sharing [CdCl_6_]^4−^ octahedra that reduced the total energy of the structure. In addition, because of the large electronegativity of chlorine, the perovskite chloride generally has higher structural stability than those of bromide and iodide. Experimentally, we studied the influence of external environment (humidity, temperature, UV light) on the optical and structural properties of CsCdCl_3_. Figure 4c shows that the as‐prepared CsCdCl_3_ SCs are still pure phase and the structural integrity is maintained rather than destroyed, after storing a long period of time for 50 days in air (RT, 75% humidity). Moreover, the initial emission performance of sample can be efficiently maintained after 50‐day storage, only showing a small decay of about 11% (Figure 4d), indicating that the luminescence characteristic of CsCdCl_3_ SCs is stable even affected by moisture and oxygen. Further, the thermal stability test of CsCdCl_3_ SCs was conducted to assess its temperature tolerance. The thermogravimetric analysis (TGA) displayed in Figure S12, Supporting Information, evidence that CsCdCl_3_ is thermally stable up to a high temperature of 550 °C. Besides, time‐dependent PL spectra of CsCdCl_3_ SC under continuous heating at 100 °C were performed. Figure 4e presents a pseudocolor map of the time‐dependent PL spectra for 10 h, over which the CsCdCl_3_ SC was continuously excited by UV light. Clearly, the emission properties of sample are very robust over the entire test with the spectral shape, peak intensity, and peak position almost unchanged. Subsequently, the sample was employed for successive five‐cycled LPL decay test. As displayed in Figure 4f, the persistent luminescence decay trend of CsCdCl_3_ SC can be well maintained over five cycles of re‐activation, evidencing the outstanding LPL stability of CsCdCl_3_. Overall, the results obtained indicate that CsCdCl_3_ has a good stability under the influence of moisture, oxygen, heat, and light, proving the reliable capability of this LPL material for practical applications in harsh conditions.

**Figure 5:**
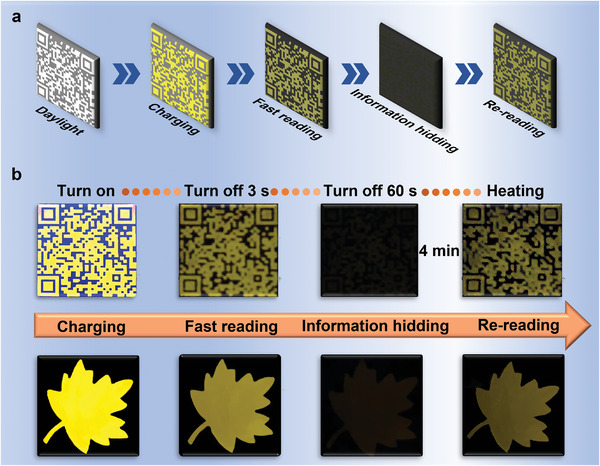
a) Schematic of dual‐mode information storage‐reading method. The prepared pattern is charged under an UV lamp at 254 nm for 5 min. When the information is not recognized, the pattern is heated to 180 °C for re‐reading. b) Dual‐mode information storage‐reading photographs of QR code and maple leaf patterns.

Considering its attractive fluorescence and persistent luminescence properties as well as outstanding stability, the as‐prepared CsCdCl_3_ SCs are applied for information storage. **Figure** **5**a shows a dual‐mode information storage‐reading method we designed. First, we use a 254 nm lamp to excite the prepared pattern for 5 min, and the photo‐generated electrons are trapped by trapping centers in CsCdCl_3_ to store energy as described in Figure 3g, which can be deemed as the charging process. Second, the pattern information is identified due to the LPL of CsCdCl_3_ at RT when the lamp is removed, because the carriers in shallow traps are released to the luminescent centers. This process is called fast reading (stage I in Figure 3d). Third, after removing the excitation light for 1 min, the electrons in the shallow traps are almost emptied, thus the emission intensity diminishes substantially and the information is gradually hidden, resulting in the un‐recognition of pattern information. Finally, by placing the pattern at a high temperature (180 °C) for 4 min, the information reappears, which is due to that the charge carriers in the deep traps obtain enough thermal energy to enable the de‐trapping process and reach the emission centers (stage III in Figure 3d). Thus, information re‐reading is achieved. Experimentally, the large‐sized CsCdCl_3_ SCs were ground into powder and then filled into different molds to prepare patterns with different information. Figure S13, Supporting Information, shows the photos of the as‐prepared flat patterns of quick response (QR) code and leaf in natural light with the pattern size of 4 cm × 4 cm. Figure 5b exhibits the information storage‐reading process for QR code and leaf patterns. The QR code pattern emits yellow fluorescence under UV light excitation. After stopping UV excitation to end the charging process, the QR code exhibits yellow LPL emission. The website homepage can be conveniently identified by scanning the LPL QR code with a handphone, to realize the information reading.^[^ ^3^ ^]^ Gradually, the LPL is too weak for the QR code information to be recognized. Extraordinarily, the information of QR code is re‐read by heating the pattern. Similarly, the leaf pattern exhibits a yellow LPL after ceasing UV light excitation, and the pattern information can be re‐read when the invalid pattern is heated to 180 °C. Therefore, dual‐mode information storage‐reading is achieved thanks to the LPL characteristics of CsCdCl_3_ SC, implying the application prospect of CsCdCl_3_ in the field of information storage.

## 3 Conclusion

In conclusion, we demonstrated a high‐performance LPL material based on an undoped inorganic metal halide CsCdCl_3_ synthesized by the facile hydrothermal method. Under UV light excitation, the CsCdCl_3_ SCs exhibit a broadband yellow emission with a PLQY as high as ≈90%, and the RT LPL emission lasts up to 6000 s. Joint experiment‐theory characterizations suggest that the carrier de‐trapping from shallow and deep trap sites induced by the intrinsic point defects in CsCdCl_3_ to localized emission centers contributes to the LPL. Moreover, the CsCdCl_3_ SCs demonstrate good optical and structural stability under the influence of moisture, oxygen, heat, and light. Even after 50 days of storage in 75% humidity air or 10 h heating at 100 °C, the CsCdCl_3_ SCs can still maintain LPL luminescence characteristics, manifesting a reliable LPL material compatible for practical application. Encouraged by the attractive fluorescence and persistent luminescence properties as well as outstanding stability of CsCdCl_3_ SCs, we successfully implemented a dual‐mode information storage‐reading application. The results obtained represent a groundbreaking advance in the fields of undoped metal halides LPL materials, highlighting the great potential in information storage applications.

## 4 Experimental Section

### Materials

Cadmium chloride (CdCl_2_, >99.99%), cesium chloride (CsCl, >99.9%), hydrochloric acid (HCl, 36 wt.% in water), and ethyl alcohol were purchased from Aladdin Chemistry Co., Ltd. (China).

### Synthesis of CsCdCl_3_ Crystals

The CsCdCl_3_ crystals with a millimeter size were prepared by hydrothermal technique. Specifically, 2 mmol of CsCl, 2 mmol of CdCl_2_, and 5 mL concentrated HCl were added to a 25 mL Teflon liner. The solution was then heated to 180 °C in the stainless steel Parrautoclave, and then maintained at this temperature for 12 h to fully dissolve the raw materials. Following that, the temperature of drying chamber was slowly cooled to RT at the rate of 2.5 °C h^−1^. Then, the unwanted hydrochloric acid solution was removed with a rubber tipped dropper, and the precipitated crystals were washed three times with ethylalcohol to remove the impurity. Finally, the prepared CsCdCl_3_ crystals were dried in a drying chamber (12 h at 80 °C).

### Preparation of Information Storage Patterns

Different patterns were prepared through two steps: first, the CsCdCl_3_ SCs were ground into small‐sized powders with a grinding bowl; second, the obtained powders were filled into the customized pattern templates; finally the patterns were scraped and pressed by a glass slide to get flat patterns in the shapes of leaves or QR code.

### Materials and Characterizations

The structural characterizations of CsCdCl_3_ crystals were examined by XRD (TD‐3500, China). The morphology and chemical compositions were observed by SEM (JEOL, JSM‐7500F) and EDS. The microstructure was further characterized by TEM (JEOL, JEM‐3010). Absorption spectrum was performed by an UV–vis spectrophotometer (UV–vis, Hitachi UH4150). PL/PLE spectra were monitored by the fluorescence spectrophotometer (Horiba; Fluorolog‐3). The PLQY of the CsCdCl_3_ crystals was performed in FLS‐1000 spectrometer with a calibrated integrating sphere. The transient PL spectrum of CsCdCl_3_ crystals was measured by the time‐resolved spectrometer (Delta‐flex, Horiba Scientific). TGA was obtained successfully by TGA Q5000IR under the N_2_ atmosphere. The LPL decay curve was monitored at 580 nm by thermoluminescence spectrometer (TOSL‐3DS). When the remained signal level was two times to that of background, the corresponding time was considered as the LPL decay time. The samples were charged with a 254 nm lamp for 5 min before transferring into a heating chamber. TOSL‐3DS thermoluminescence spectrometer was used to obtain the thermoluminescence spectrum at a heating rate of 1 K s^−1^ (Guangzhou Rongfan Science and Technology Co., Ltd).

### Theoretical Calculations

According to density functional theory (DFT), the first‐principles calculations were conducted. The Vienna Ab initio Simulation Package (VASP) was used to implement the plane‐wave pseudopotential method. Projector augmented wave (PAW) approach was chosen and treated 5s^2^5p^2^6s for Cs, 4d^10^5s^2^ for Cd, and 3s^2^3p^5^ for Cl as valence electrons. The DOS and electronic band structures were calculated with Perdew–Burke–Ernzerhof (PBE) exchange‐correlation functional. The canonical (NVT) ensemble with the Nosé thermostat was used to conduct the molecular dynamics simulations at 300 K, and the last time was 6 ps while the time step was 1 fs.

The formation energy ∆H of point defects was calculated according to the following equation:

(1) $$\Delta H=\left(E_{D}-E_{H}\right)-\sum_{i}n_{i}\left(\mu_{i}+\mu_{i}^{\mathrm{bulk}}\right)+q\left(\varepsilon_{\mathrm{VBM}}+\varepsilon_{f}\right)+\Delta E_{\mathrm{corr}}$$

in which, *E*_D_ is the total amount of energy of supercells that contain impurities. *E*_H_ is total amount of energy of the impurity‐free supercells. *n_i_* is a difference number of atoms for the ith atomic species between the defect‐containing and defect‐free supercells. *µ_i_* is the relative chemical potential for the ith atomic species referenced to its bulk $\mu_{i}^{\mathrm{bulk}}$, *ε*_VBM_is the energy of the VBM of the host material, and *ε*_*f*_ is the Fermi energy relative to the VBM. The 2×2×2 supercell including 48 formula units of CsCdCl_3_ and the 2×2×1 grid for *k*‐point sampling of the Brillouin zone were adopted to perform defect formation energy calculations. A finite‐size correction was used for the supercell calculation to conduct the correction of potential alignment and image charge. The image charge correction used a calculated static dielectric constant of 6.5.

## Conflict of Interest

The authors declare no conflict of interest.

## Supporting information

Supporting Information

## Data Availability

The data that support the findings of this study are available from the corresponding author upon reasonable request.
