# Supplementary material for: High‐Efficiency and Stable Long‐Persistent Luminescence from Undoped Cesium Cadmium Chlorine Crystals Induced by Intrinsic Point Defects
Source: Adv Sci (Weinh). 2023 Feb 24;10(15):2207331. doi: 10.1002/advs.202207331 (PMC10214269; doi:10.1002/advs.202207331)
Supplement: Supplementary file 1 — Supporting Information [file ADVS-10-2207331-s001.pdf]

Supporting Information

High-Efficiency and Stable Long-Persistent Luminescence from Undoped Cesium Cadmium Chlorine Crystals Induced by Intrinsic Point Defects

Ruoting Yang, Dongwen Yang, Meng Wang, Fei Zhang, Xinzhen Ji, Mengyao Zhang, Mochen Jia, Xu Chen, Di Wu, Xin Jian Li, Yu Zhang, Zhifeng Shi, and Chongxin Shan*

Dr. Ruoting Yang, Dr. Dongwen Yang, Dr. Meng Wang, Dr. Fei Zhang, Dr. Xinzhen Ji, Dr. Mengyao Zhang, Dr. Mochen Jia, Dr. Xu Chen, Dr. Di Wu, Prof. Xinjian Li, Prof. Zhifeng Shi, Prof. Chongxin Shan
Key Laboratory of Materials Physics of Ministry of Education, School of Physics and Microelectronics, Zhengzhou University, Daxue Road 75, Zhengzhou 450052, China
E-mail: shizf@zzu.edu.cn
Prof. Yu Zhang
State Key Laboratory on Integrated Optoelectronics, College of Electronic Science and Engineering, Jilin University, Qianjin Street 2699, Changchun 130012, China

Keywords: cesium cadmium chloride, long-persistent luminescence, de-trapping, stability, information storage

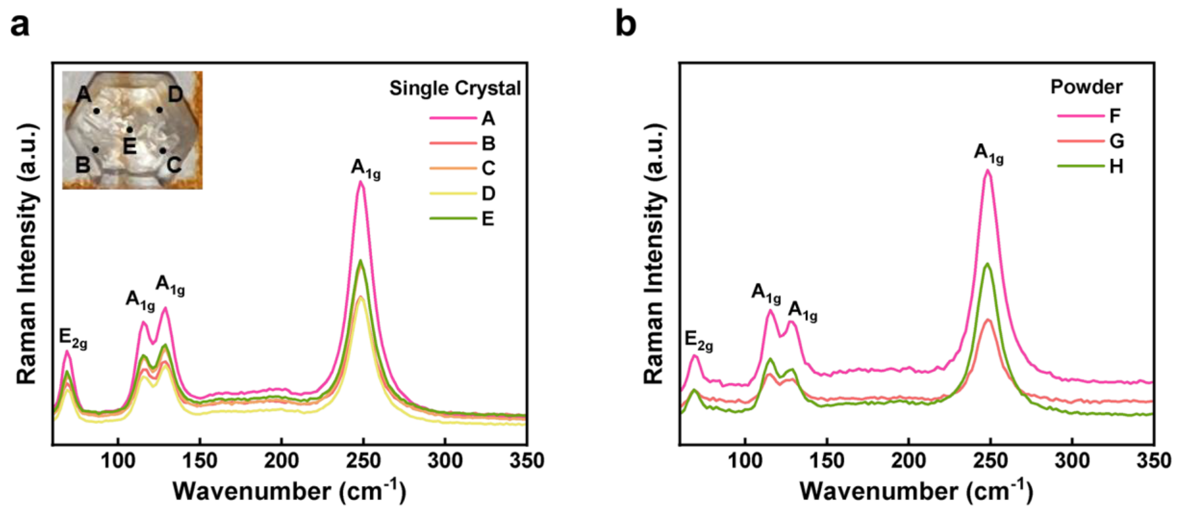

Figure S1. a) Raman spectra of $CsCdCl_3$ SC. The inset is a schematic of the selected positions. b) Raman spectra of ground powder of $CsCdCl_3$.

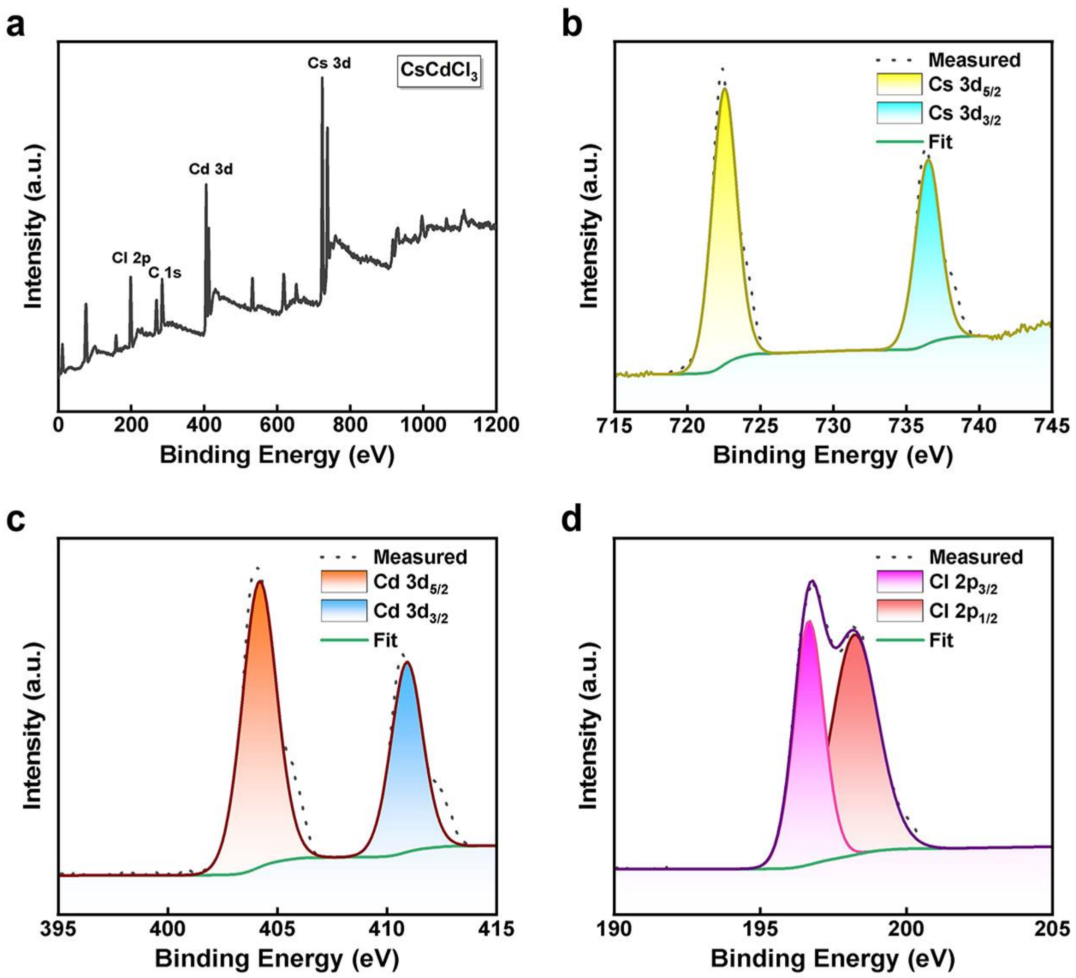

Figure S2. a) The survey XPS spectrum of $CsCdCl_3$, and the magnified regions of b) Cs 3d, c) Cd 3d, and d) Cl 2p, respectively.

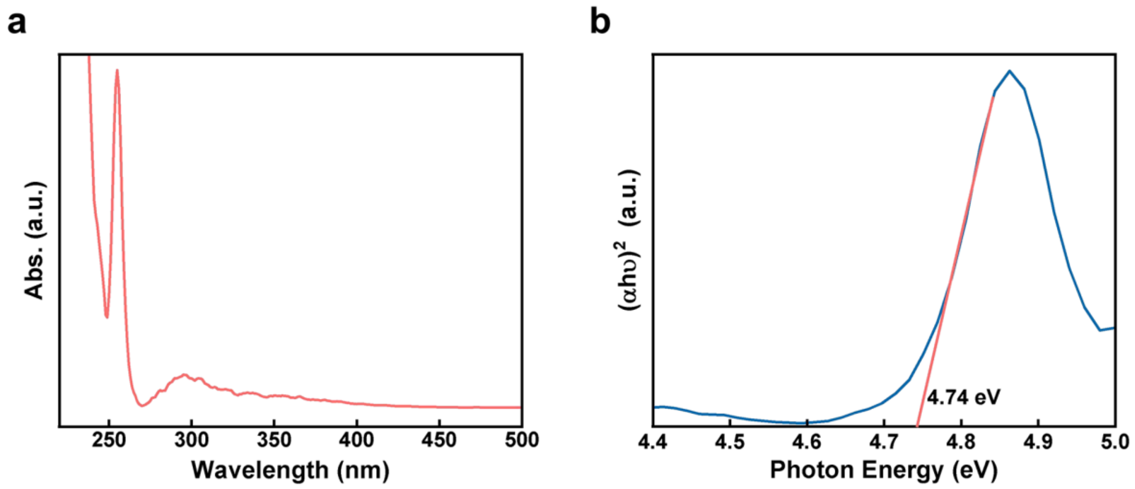

Figure S3. a) The absorption spectrum of $CsCdCl_3$ SC, and b) the corresponding Tauc plot.

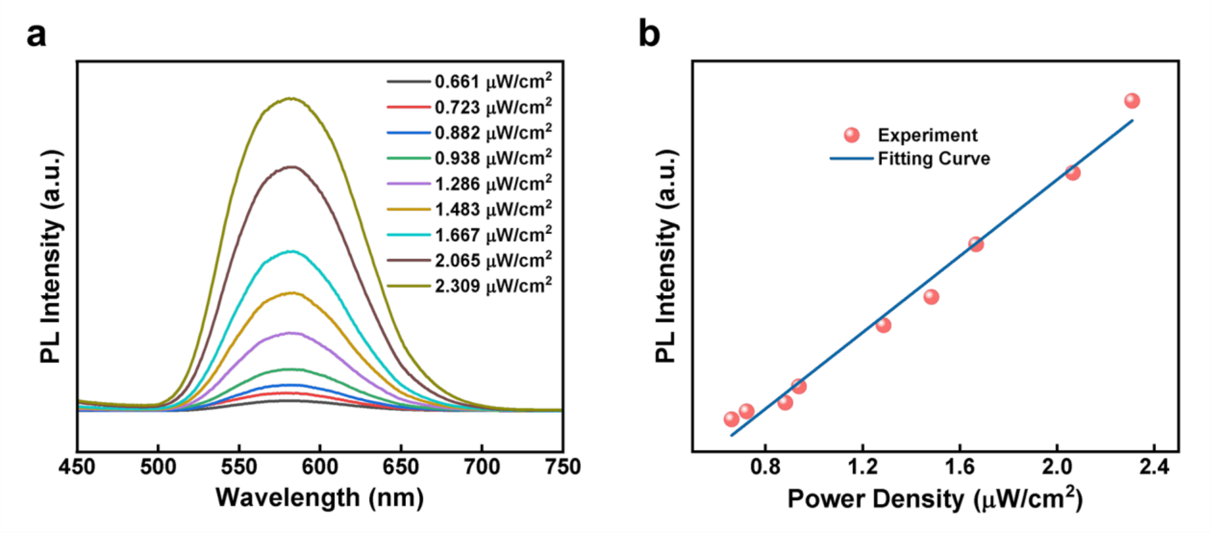

Figure S4. a) Emission spectra of $CsCdCl_3$ at different excitation powers (λ_{ex} = 254 nm). b) Excitation power-dependent PL intensity of $CsCdCl_3$.

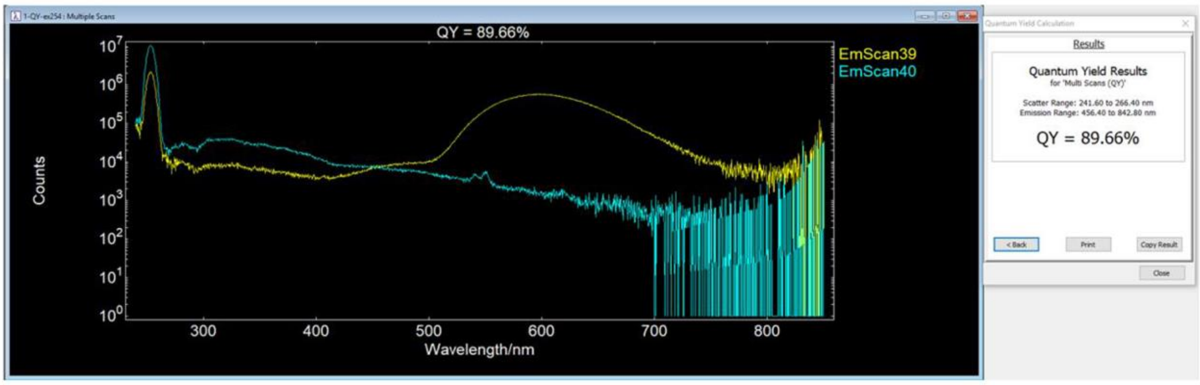

Figure S5. The measured PLQY of $CsCdCl_3$ SCs.

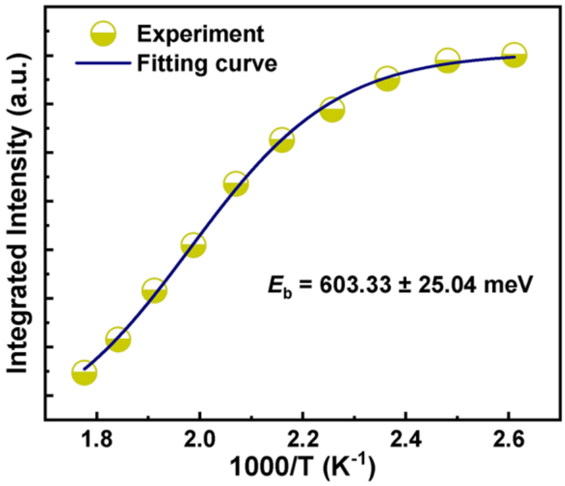

Figure S6. The integrated PL intensity as a function of reciprocal temperature.

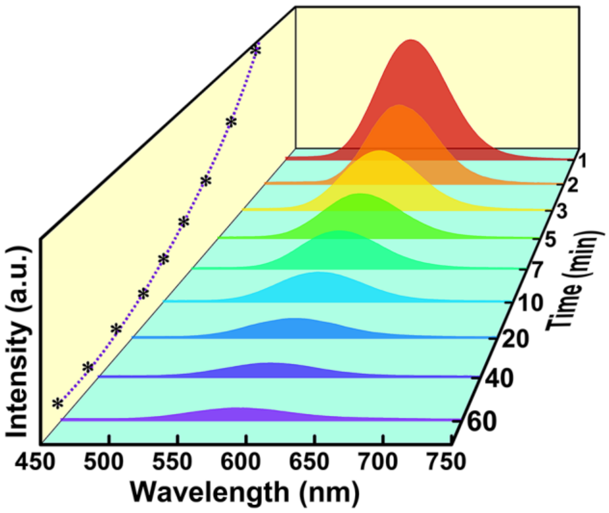

Figure S7. Time-dependent LPL spectra recorded after cessation of 254 nm UV lamp.

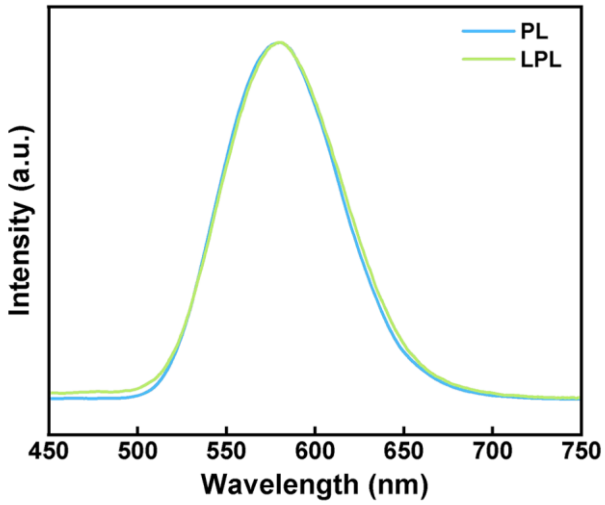

Figure S8. Normalized PL and LPL spectra of $CsCdCl_3$ SC.

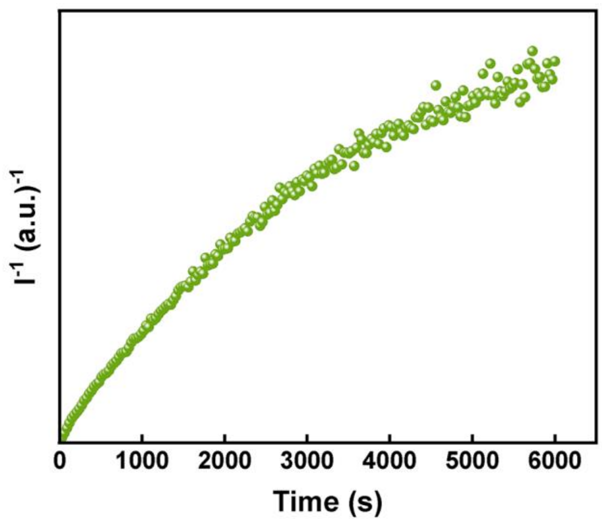

Figure S9. The light intensity-time curve ($I^{-1} - t$) converted from the LPL decay curve.

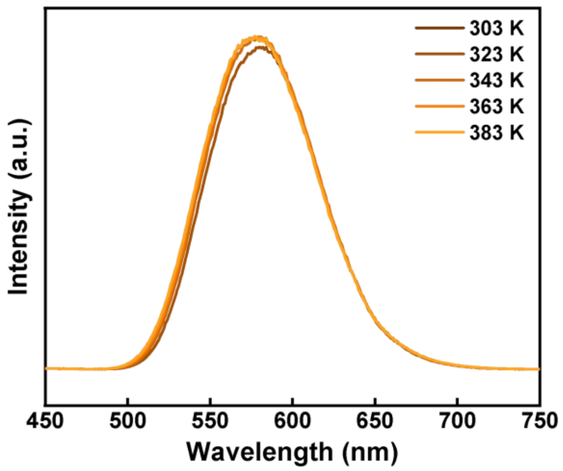

Figure S10. Temperature-dependent PL spectra of $CsCdCl_3$ in the range of 303–383 K.

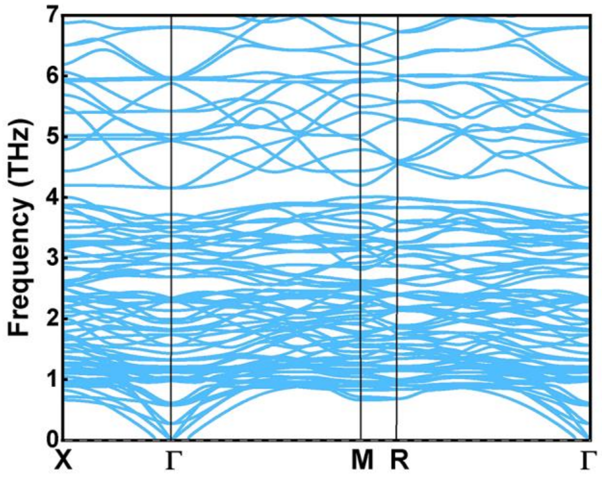

Figure S11. Phonon band structures of the $CsCdCl_3$.

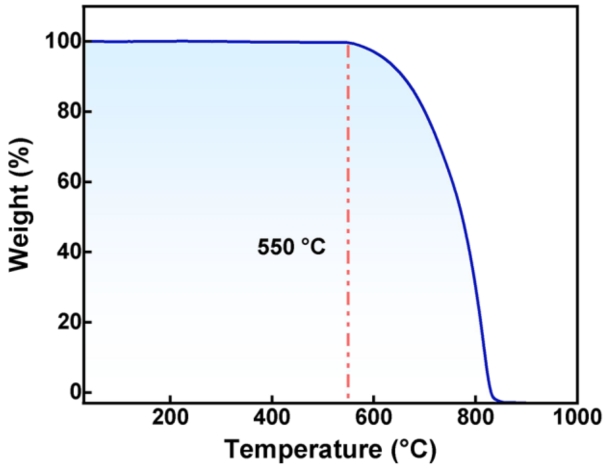

Figure S12. Thermogravimetric analysis of the $CsCdCl_3$ SC.

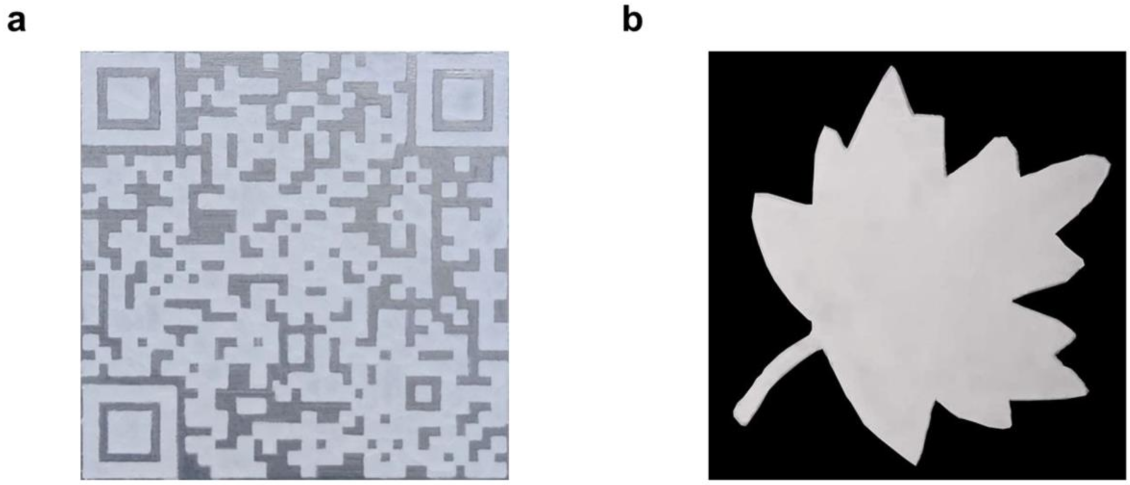

Figure S13. Pictures of as-prepared QR code and leaf patterns in natural light with the size of 4 cm × 4 cm.

Table S1. XRD data of hexagonal $CsCdCl_3$.

Compound	$CsCdCl_3$
Formula weight (g/mol)	992.9
Crystal system	Hexagonal
Space group	P63/mmc
Color	White
a (Å)	7.4030
b (Å)	7.4030
c (Å)	18.4060
α (°)	90
β (°)	90
γ (°)	120
Volume (Å^3)	90.0000
Z	6
ρ_{calc} (g/cm^3)	4.01

Table S2. Atomic positions of hexagonal $CsCdCl_3$.

Lable	x	y	z	Occupancy
Cs1	0.66667	0.33333	0.59015	1.00000
Cs2	0.33333	0.66667	0.40985	1.00000
Cs3	0.33333	0.66667	0.09015	1.00000
Cs4	0.00000	0.00000	0.75000	1.00000
Cs5	0.66667	0.33333	0.90985	1.00000
Cs6	0.00000	0.00000	0.25000	1.00000
Cd1	0.33333	0.66667	0.83948	1.00000
Cd2	0.66667	0.33333	0.16052	1.00000
Cd3	0.33333	0.66667	0.66052	1.00000
Cd4	0.00000	0.00000	0.00000	1.00000
Cd5	0.66667	0.33333	0.33948	1.00000
Cd6	0.00000	0.00000	0.50000	1.00000
Cl 1	0.16442	0.32884	0.58225	1.00000
Cl 2	0.67116	0.83558	0.58225	1.00000
Cl 3	0.16442	0.32884	0.91745	1.00000
Cl 4	0.32884	0.16442	0.08255	1.00000
Cl 5	0.67116	0.83558	0.91745	1.00000
Cl 6	0.49258	0.50742	0.75000	1.00000
Cl 7	0.16442	0.83558	0.58225	1.00000
Cl 8	0.49285	0.98516	0.75000	1.00000
Cl 9	0.16442	0.83558	0.91745	1.00000
Cl 10	0.83558	0.16442	0.41745	1.00000
Cl 11	0.83558	0.67116	0.41745	1.00000
Cl 12	0.83558	0.67116	0.08255	1.00000
Cl 13	0.83558	0.16442	0.08255	1.00000
Cl 14	0.01484	0.50742	0.75000	1.00000
Cl 15	0.32884	0.16442	0.41745	1.00000
Cl 16	0.98516	0.49258	0.25000	1.00000
Cl 17	0.50742	0.01484	0.25000	1.00000
Cl 18	0.50742	0.49258	0.25000	1.00000

Table S3. A quantified atomic ratio of $CsCdCl_3$ SC based on the EDS data.

Element	Weight%	Atomic%
Cs	7.68	0.86
Cd	5.80	0.85
Cl	5.88	2.68
